# Research Status and Hotspots of Anticancer Natural Products Based on the Patent Literature and Scientific Articles

**DOI:** 10.3389/fphar.2022.903239

**Published:** 2022-06-17

**Authors:** Junkai Shen, Jiahuan Li, Peiming Yu, Gangjun Du

**Affiliations:** School of Pharmacy, Henan University, Kaifeng, China

**Keywords:** cancer, natural product, patent analysis, mapping knowledge domain, traditional Chinese medicine

## Abstract

**Background:** The patent literature contains a large amount of information on the internal state of current industrial technologies that are not available in other literature studies. Scientific articles are the direct achievements of theoretical research in this field and can reveal how current theories in basic research have developed. In this study, the progress and status of natural anticancer products in this field were summarized, and the research hotspots were explored through the analysis of the relevant patent literature and scientific articles.

**Methods:** Patent data were retrieved from the incoPat patent retrieval database, and paper data were retrieved from the Web of Science core set and PubMed. GraphPad Prism 8, Microsoft Excel 2010, and CiteSpace 5.8.R3 were used to perform visual processing. The analyzed patent literature includes the patent applicant type, country (or region), and technical subject. The analyzed scientific article includes academic groups, subject areas, keyword clustering, and burst detection.

**Results:** A total of 20,435 patent families and 38,746 articles were collected by 4 January 2022. At present, antitumor drugs derived from natural products mainly include 1) apoptosis inducers such as curcumin, gallic acid, resveratrol, Theranekron D6, and gaillardin; 2) topoisomerase inhibitors such as camptothecins, scaffold-hopped flavones, podophyllotoxin, oxocrebanine, and evodiamine derivatives; 3) telomerase inhibitors such as camptothecin and isoquinoline alkaloids of *Chelidonium majus*, amentoflavone, and emodin; 4) microtubule inhibitors such as kolaflavanone, tanshinone IIA analog, eugenol, and millepachine; 5) immunomodulators such as fucoidan, myricetin, bergapten, and atractylenolide I; 6) tumor microenvironment regulators such as beta-escin and icaritin; 7) multidrug resistance reversal agents such as berberine, quercetin, and dihydromyricetin; and 8) antiangiogenic and antimetastatic agents such as epigallocatechin-3-gallate, lupeol, ononin, and saikosaponin A.

**Conclusion:** Anticancer natural product technology was introduced earlier, but the later development momentum was insufficient. In addition, scientific research activities are relatively closed, and technical exchanges need to be strengthened. Currently, the development of medicinal plants and the research on the anticancer mechanism of natural active products are still research hotspots, especially those related to immune checkpoints, essential oils, and metastatic cancer. Theories of traditional Chinese medicine (TCM), such as “restraining excessiveness to acquire harmony,” “same treatment for different diseases,” “Meridian induction theory,” and “Fuzheng Quxie,” have important guiding significance to the research of anticancer mechanisms and the development of new drugs and can provide new ideas for this process.

**Systematic Review Registration**: [https://sourceforge.net/projects/citespace/], identifier [000755430500001].

**Systematic Review Registration:** [website], identifier [registration number].

## Introduction

A total of 1,918,030 new cancer cases and 609,360 cancer-related deaths are expected to occur in the United States in 2022, according to a cancer statistics report from the American Cancer Society (Siegel et al., 2022), and statistics also show that more than 60% of the currently approved cancer drugs or drug candidates come from natural sources (Ahuja et al., 2020). In addition, the broad spectrum of natural products provides lead compounds amenable to structural modification; their discovery greatly enriches the selection of anticancer compounds, and their multitarget, broad spectrum characteristics, and low toxicity have made them increasingly prominent in cancer prevention and treatment. We also believe that with the increasing cost of Western medicine research and development and the lengthy process, therapies using natural drugs will become the most promising type of anticancer drug.

As a common malignant process, cancer is characterized by cells with biological characteristics such as cell differentiation, abnormal proliferation, uncontrolled growth, invasion, and metastasis, and its occurrence is often a complex process involving multiple factors and steps. Natural medicines can be divided into drugs with direct action and indirect action, according to their biological characteristics and action targets. The first category includes drugs that act directly on tumor cells. For example, many natural drugs induce apoptosis by regulating the expression of key apoptotic proteins, thereby inhibiting tumor growth (Wang et al., 2018); some natural products, such as camptothecin, act on topoisomerase targets and prevent DNA replication in tumor cells by inhibiting the topoisomerase activity (Ottaviani et al., 2021); taxanes (Thakkar et al., 2021) and periwinkle alkaloids (Celik et al., 2017) interfere with microtubule formation by binding to microtubules and thus display anticancer properties. Other indirect ways to prevent cancer development are to improve the tumor microenvironment (Zhu et al., 2020), inhibit tumor angiogenesis and cancer cell invasion or adhesion (Lei et al., 2020), reverse multidrug resistance of tumor cells (Neophytou et al., 2021), or activate immune cells to regulate the body’s immune function (Wayne et al., 2021). James Allison’s team was awarded the 2018 Nobel Prize in physiology or medicine for pioneering the concept of “immune checkpoint” and demonstrating for the first time that the CTLA-4 antibody enhances immunosuppression of tumor development in mice, making immunotherapy a promising treatment for cancer.

With the advance in analytical methods and the emergence of new analytical instruments, the amount of basic research on natural products in the field of cancer treatment has been greatly increasing. In recent years, the volume of the relevant patent literature and scientific articles has also shown considerable growth. It is not easy to understand the development and status of natural anticancer drugs from such a variety of information. Based on the patent literature and scientific articles, bibliometric analysis was conducted from two aspects of technical and theoretical research to provide a broad perspective for understanding the research hotspots and status quo in this field.

## Methods

### Data Source

The incoPat 6.0 patent retrieval database was queried with the following search string: [TIAB=(“natural product*” or “natural medicine*” or “natural drug*” or (Chinese medicine*) or “herb*” or “natural compound*” or “natural molecule*” or “phytochemical*” or 天然 or 中药 or 草药 or 植物)] and [IPC=(A61P35/00)].

The Web of Science core collection online database was queried with the following search string: TS=(“natural product*” or “natural medicine*” or “natural drug*” or (Chinese medicine*) or “herb*” or “natural compound*” or “natural molecule*” or “phytochemical*”) and TS=(“cancer*” or “tumor*” or “neoplasm*”).

**FIGURE 1:**
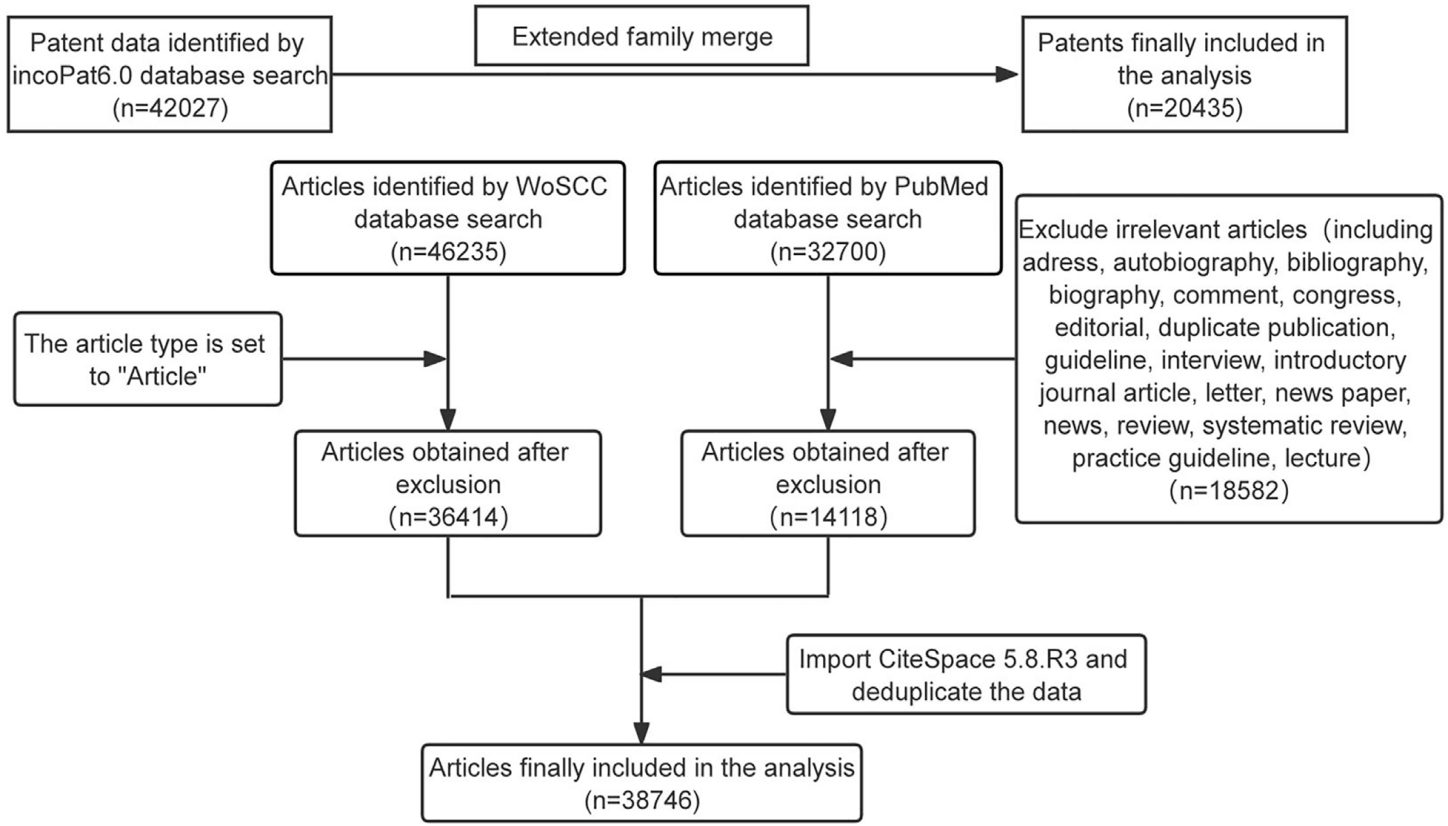
Flow chart of data collection.

The PubMed citation database was queried with the following search string: {[“natural product*” (Title/Abstract) or “natural medicine*” (Title/Abstract) or “natural drug*” (Title/Abstract) or [Chinese medicine*(Title/Abstract)] or “herb*” (Title/Abstract) or “natural compound*” (Title/Abstract) or “natural molecule*” (Title/Abstract) or “phytochemical*” (Title/Abstract)]} and [“cancer*” (Title/Abstract) or “tumor*” (Title/Abstract) or “neoplasm*” (Title/Abstract)]. The time frame deadline for all the aforementioned data retrievals was 4 January 2022. The flow chart of data collection is shown in Figure 1.

### Data Collection and Analysis

All patent data and scientific article data were downloaded by the author (JK Shen) independently from the incoPat 6.0 patent database, Web of Science core set, and PubMed. The analyzed patent literature includes the patent applicant type, country (or region), and technical subject. The analyzed scientific article includes academic groups, subject areas, keyword clustering, and burst detection.

Visualization of the patent literature is based on tools, GraphPad Prism 8 and Microsoft Excel 2010, and the visualization of scientific articles is mainly based on CiteSpace5.8.R3. CiteSpace parameters were set as follows: the time slice was from 1984 to 2021, and the cutting life (#years per slice) was set to one, a cosine algorithm was used to calculate the relation strength (link), selection criteria selected the “TOP N” algorithm for extraction, and the N value was set to 50. Other parameters were left in their initial state. The LLR (log-likelihood Rate) algorithm was used for clustering analysis in this study.

## Results

### Annual Patent Application Trend

**FIGURE 2:**
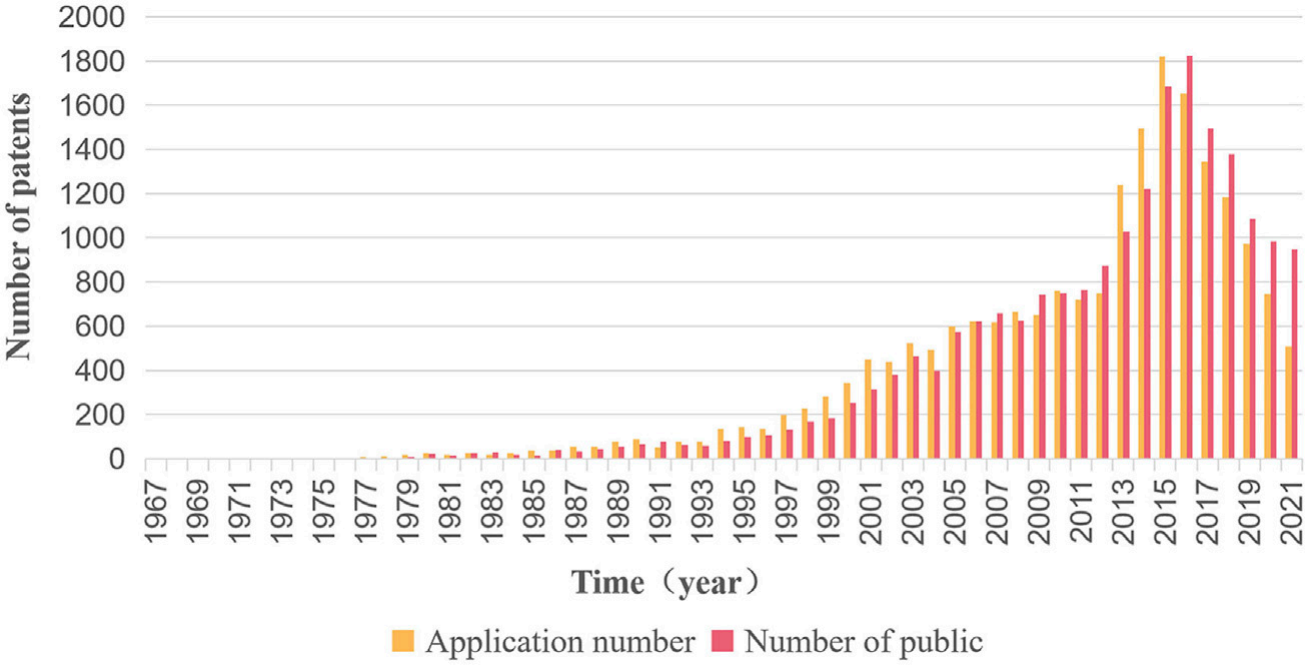
Patent application trends of anticancer natural products. The number of patent applications peaked in 2015.

The analysis of the annual patent application trend can clearly show the changes in the patent layout of anticancer natural products in various periods from a macro level. The statistical results are shown in Figure 2.

The number of patent applications for natural products in the field of cancer treatment showed an overall rising trend, and the total number of patent applications reached 20,435 by 2 January 2022. Patent applications began in 1967, and the number of patent applications in that year was only three, while in 2005, patent applications began to grow rapidly, and the average growth rate of patent applications this time reached 20.29%. From 2006 to 2012, the number of patent applications tended to be stable and peaked in 2015.

### Application Geographical Distribution

The analysis of application geographical distribution can show the status of technological innovation in different countries and identify the countries that are the main source and important target markets for technological innovations.

**FIGURE 3:**
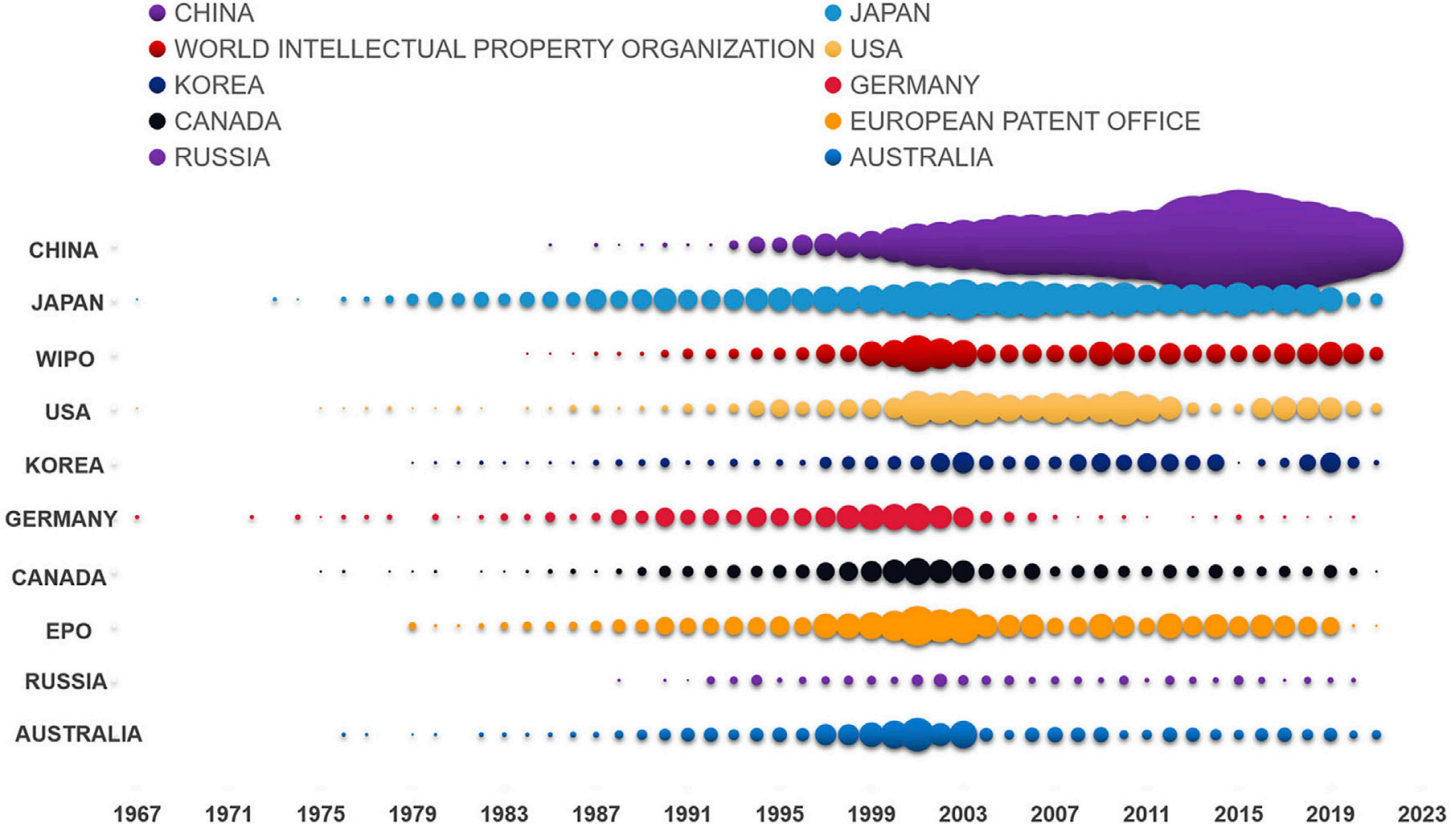
Annual patent application trend of the top 10 countries or organizations in the number of anticancer natural product patent applications. Patent applications began in Japan, the United States, and Germany. The bubbles in the figure reflect the evolution trend of the number of patent applications of each country or organization in the time dimension.

**FIGURE 4:**
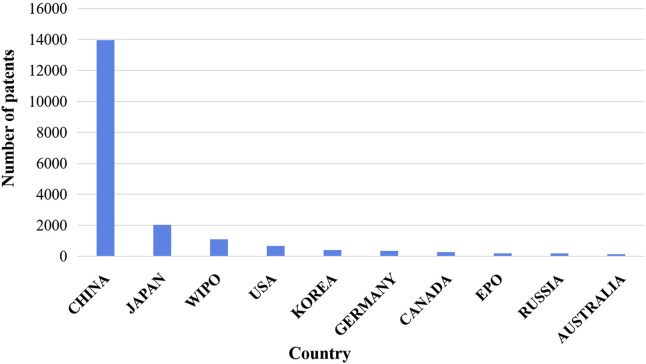
Distribution of the top 10 countries or organizations in the number of patent applications for anticancer natural products. China ranked first in the number of disclosed patents for natural anticancer drugs. WIPO, World Intellectual Property Organization; EPO, European Patent Office.

As shown in Figure 3, patent applications began in Japan, the United States, and Germany. At present, patents for natural anticancer products are distributed in 75 countries, regions, or organizations. China ranked first in the number of disclosed patents for natural anticancer drugs with 13,967, accounting for 68.35% of the global total. This number was followed by the number of patents in Japan (9.90%, with 2024 patents), the World Intellectual Property Organization (5.43%, with 1,109 patents), the United States (3.23%, with 660 patents), South Korea (1.94%, with 396 patents), Germany (1.76%, with 359 patents), Canada (1.25%, with 256 patents), EPO (0.94%, with 256 patents), Russia (0.92%, with 187 patents), and Australia (0.62%, with 126 patents) (Figure 4).

### Applicant Distribution

The incoPat database reflects the value of each patent by scoring each patent from three aspects: technical stability, technological advancement, and protection scope. Therefore, it can automatically evaluate the patent strength of each patent and obtain high-value patents quickly. Here, we screened patents with a value greater than five points for statistics. This analysis can find the patent applicants with more innovation achievements and further reflect the patent competitive strength of each applicant.

**FIGURE 5:**
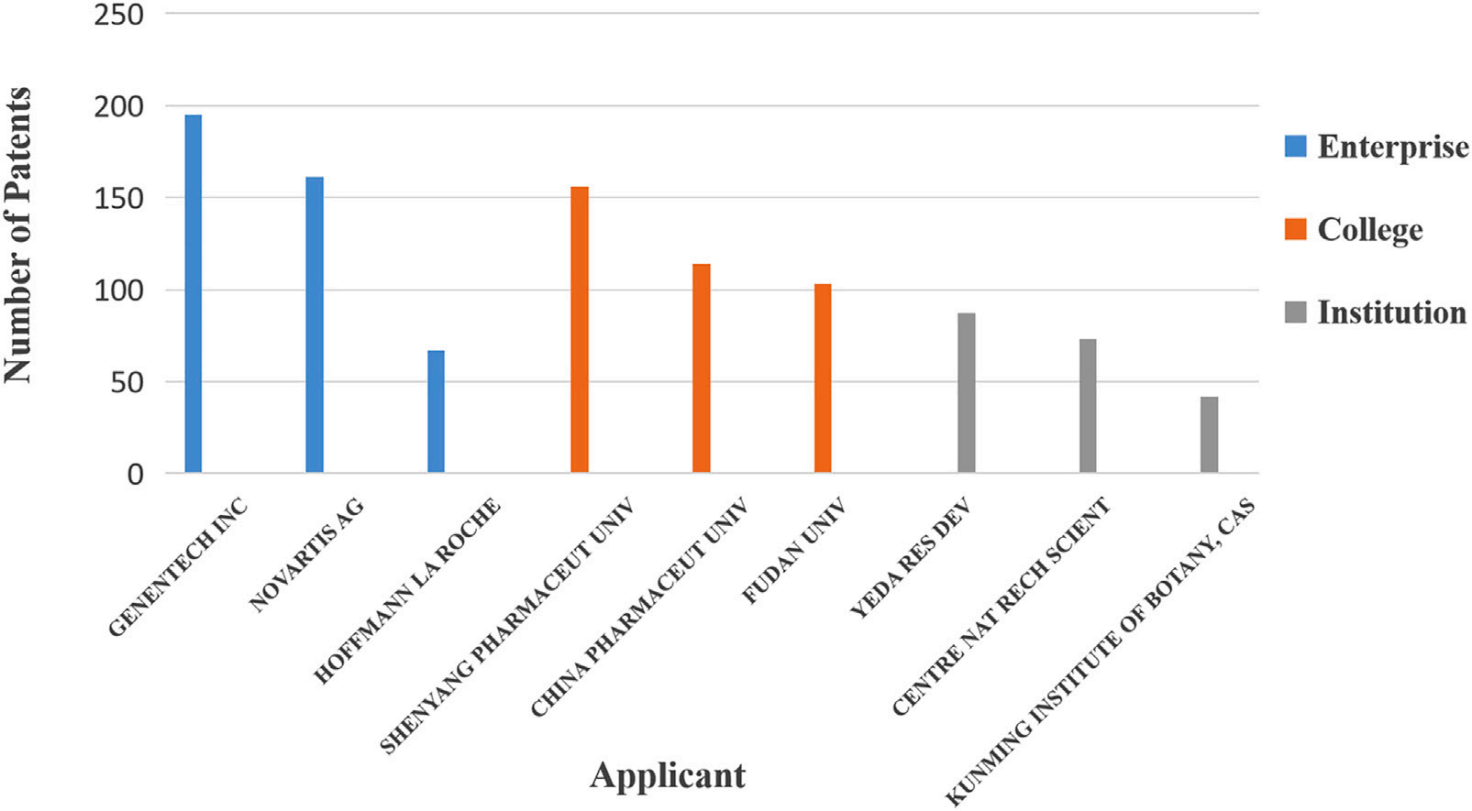
Distribution of high-value patents for anticancer natural products based on the type of applicant. Genentech, Inc., has accumulated the largest number of patents in this field.

As shown in Figure 5 and Table 1, Genentech, Inc., Novartis AG, and Hoffmann-La Roche have accumulated a large number of high-value patents in the field of natural anticancer drugs, among which Hoffmann-La Roche has the highest average patent value. Based on the distribution of patents in universities, Shenyang Pharmaceutical University, China Pharmaceutical University, and Fudan University in China occupy the leading position, and those from China Pharmaceutical University have the highest average patent value. Among scientific research institutions, Yeda Research and Development, Centre National de la Recherche Scientifique, Kunming Institute of Botany, and the Chinese Academy of Sciences are the leading forces in patent applications in this field, and Yeda Research and Development has the highest average value.

**TABLE 1:** Distribution list of high-value patents of various types of applicants for anticancer natural products.

Applicant	Country	Value degree					Amount	Average
		6	7	8	9	10		
Genentech, Inc.	United States	1	10	22	17	145	195	9.51
Novartis AG	Switzerland	1	7	18	27	108	161	9.45
Hoffmann La-Roche	Switzerland	0	3	2	19	43	67	9.52
Shenyang Pharmaceutical University	China	23	18	56	49	10	156	8.03
China Pharmaceutical University	China	17	23	16	46	12	114	8.11
Fudan University	China	30	43	16	13	1	103	7.15
Yeda Research and Development	Israel	1	1	3	19	63	87	9.63
Centre National de la Recherche Scientifique	France	1	3	10	11	48	73	9.40
Kunming Institute of Botany, CAS	China	4	11	6	17	4	42	8.14

### Composition of Patented Technology

Patent examiners assign each patent to different IPC classification numbers based on the technical field involved. This analysis can show the technology categories covered by the analysis object and the innovation enthusiasm of each technology branch.

**FIGURE 6:**
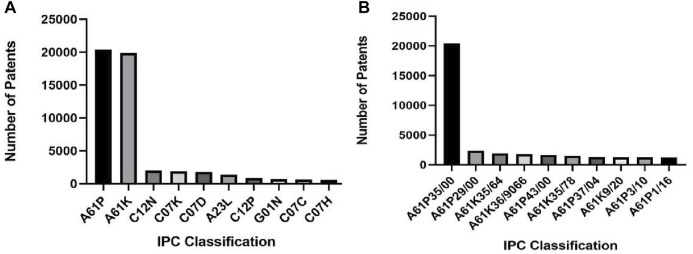
Patented technology distribution of anticancer natural products. The IPC classification number reflects the technical field covered by each patent. **(A)** Technology distribution on the IPC subclass dimension. Anticancer natural products are mainly distributed in the A61P and A61K subclasses. **(B)** Technology distribution on IPC group dimension. Anticancer natural products are mainly distributed in the A61P35/00 and A61P29/00 groups.

As shown in Figure 6, 1) anticancer natural products are mainly distributed into A61P, A61K, C12N, C07K, C07D, C12P, G01N, C07K, A23L, C07C, and C07H subclasses; 2) from the dimension analysis of the IPC group, except for the A61P35/00 group, patents are mainly distributed into A61P29/00, A61K35/64, A61K36/9066, A61P43/00, A61K35/78, A61K9/20, A61P3/10, A61P37/04, and A61P1/16 technical branches. The specific meaning of IPC classification number is shown in Table 2.

**TABLE 2:** IPC classification number technical description.

IPC (subclass)	IPC (group)
A61P: specific therapeutic activity of chemical compounds or medicinal preparations	A61P35/00: antineoplastic agents
A61K: preparations for medical, dental, or toilet purposes	A61P29/00: non-central analgesic, antipyretic, or anti-inflammatory agents, for example, anti-rheumatic agents and non-steroidal anti-inflammatory drugs (NSAIDs)
C12N: microorganisms or enzymes; compositions thereof; propagating, preserving, or maintaining microorganisms; mutation or genetic engineering; and culture media	A61K35/64: insects such as bees, wasps, or fleas
C07K: peptides	A61K36/9066: Curcuma, for example, common turmeric, East Indian arrowroot, or mango ginger
C07D: heterocyclic compounds	A61P43/00: drugs for specific purposes, not provided for in groups A61P1/00–A61P41/00; classification is only made in this group when a specific therapeutic activity for a chemical compound or medicinal preparation has been clearly disclosed, the specific therapeutic activity not being appropriate to any of groups A61P1/00–A61P41/00
A23L: foods, foodstuffs, or non-alcoholic beverages, not covered by subclasses A21D or A23B–A23J; their preparation or treatment, for example, cooking, modification of nutritive qualities, and physical treatment; and preservation of foods or foodstuffs, in general (preservation of flour or dough for baking A21D)	A61K35/78: transferred to A61K36/00, medicinal preparations of undetermined constitution containing material from algae, lichens, fungi, or plants, or derivatives thereof, for example, traditional herbal medicines
C12P: fermentation or enzyme-using processes to synthesize a desired chemical compound or composition or to separate optical isomers from a racemic mixture	A61P37/04: immunostimulants
C07C: acyclic or carbocyclic compounds	A61K9/20: pills, lozenges, or tablets
G01N: investigating or analyzing materials by determining their chemical or physical properties	A61P3/10: for hyperglycaemia, for example, antidiabetics
C07H: Sugars; derivatives thereof; nucleosides; nucleotides; and nucleic acids	A61P1/16: for liver or gallbladder disorders, for example, hepatoprotective agents, cholagogues, and litholytics

### Annual Trend of Scientific Articles

Usually, scientific articles are an intuitive manifestation of the basic theoretical research results in the subject area. Through the publication trend of annual scientific articles, we can understand the development trend of scientific research and innovation activities in the field of anticancer natural products from a macro perspective.

**FIGURE 7:**
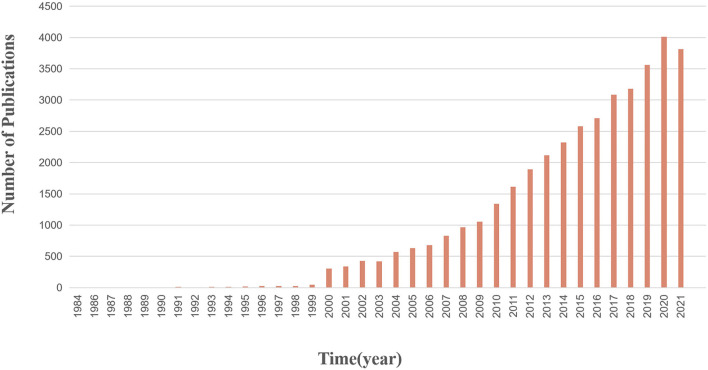
Annual trend chart of scientific articles in the field of anticancer natural products. The publication of scientific articles first started in 1984 and reached a peak of 4,013 in 2020.

As shown in Figure 7 **,** 38,746 articles have been published in this field, spanning 39 years. The publication of scientific articles started in 1984 and reached a peak of 4,013 in 2020. Overall, the number of articles published shows an upward trend, indicating that scientific research and innovation activities tend to be active.

### Research Cooperation Network

There are many forms of scientific research cooperation, and the scientific research cooperation mentioned in this article refers to the countries or scientific research units that appear at the same time in an article, and thus, we determined that there is a cooperative relationship between them. Through this analysis, we can understand the main academic groups and academic cooperation of basic theoretical research in this field and provide references for the introduction or cooperation of academic resources in the future.

**FIGURE 8:**
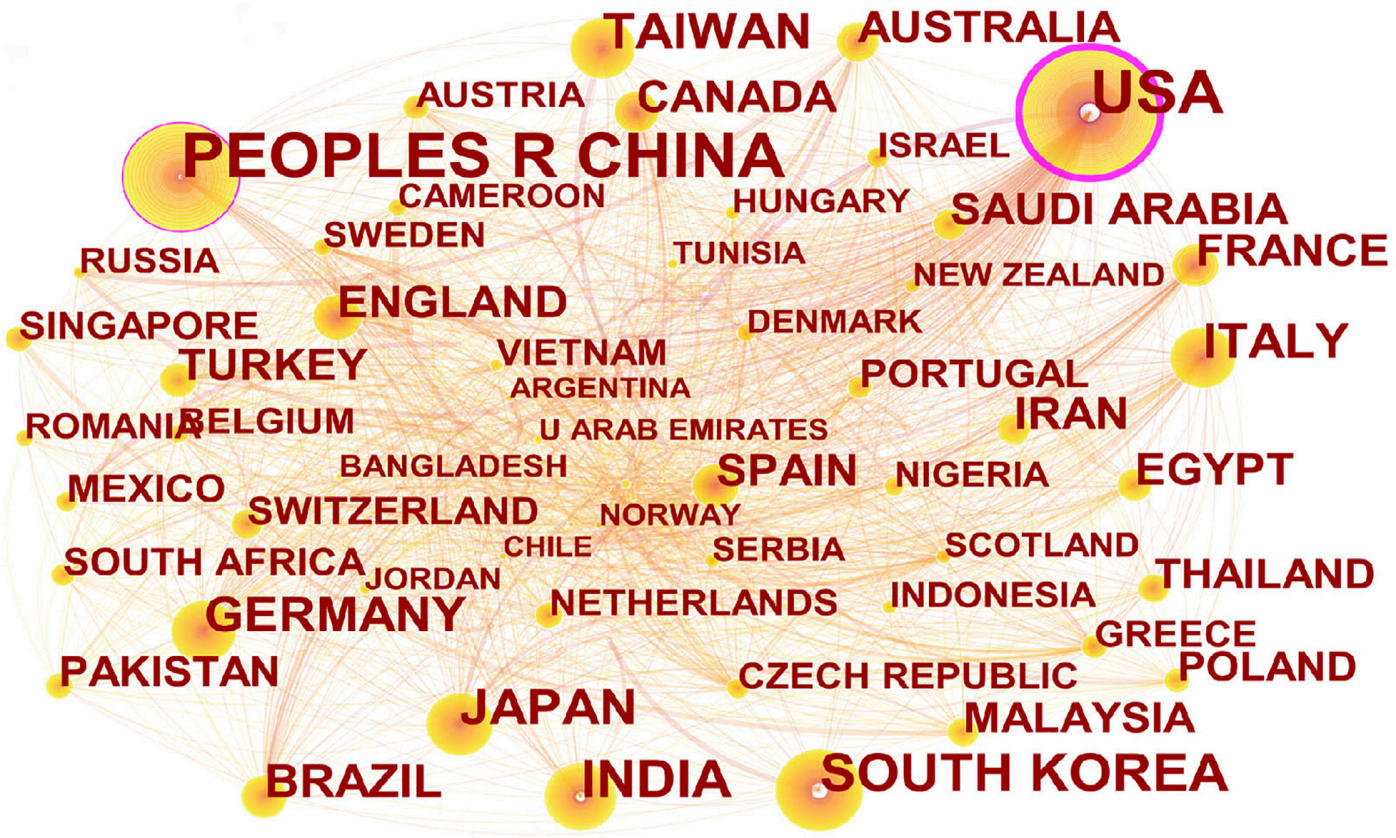
National or regional cooperation network. China ranks first in the number of articles published; the United States and China have higher centrality. The size of the nodes represents the number of articles published by the country, the lines between the nodes represent the cooperative relationship, and the thickness of the lines represents the co-occurrence strength. Nodes with centrality greater than 0.1 are marked with a purple outer circle.

**FIGURE 9:**
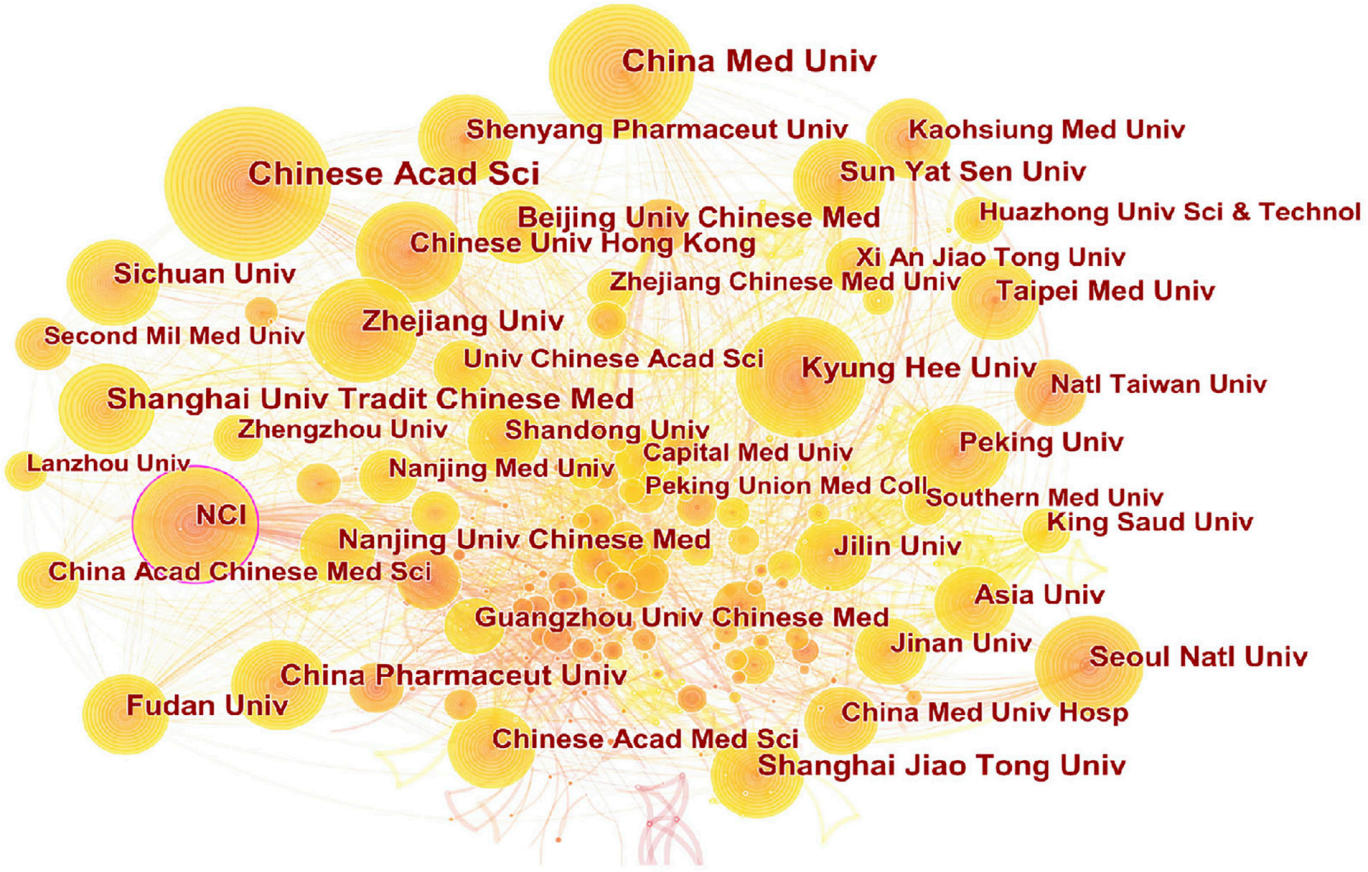
Cooperation network of scientific research institutions. Chinese Academy of Sciences ranks first in the number of articles published; the National Cancer Institute of the United States (NCI) has the highest centrality. The size of the nodes represents the number of articles published by the scientific research institutions, the lines between the nodes represent the cooperative relationship, and the thickness of the lines represents the co-occurrence strength. The larger the node, the greater is the number of articles. Nodes with centrality greater than 0.1 are marked with a purple outer circle.

As shown in Figure 8, China ranked first with 13,323 articles published, followed by the United States, India, and South Korea. From the point of view of node centrality, the United States and China have higher betweenness. Figure 9 showed that the Chinese Academy of Sciences ranked first with 932 articles, followed by China Medical University, Shanghai University of Traditional Chinese Medicine, and Zhejiang University. In addition, the National Cancer Institute of the United States (NCI) has the highest centrality and is at the key node position in the network, followed by the Chinese Academy of Sciences.

CiteSpace5.8.R3. uses centrality to discover and measure the importance of nodes. Nodes with centrality greater than 0.1 are marked with a purple outer circle. Nodes with high centrality are usually regarded as key hubs connecting two different fields, also called “turning points.” From the perspective of information transmission, the higher the centrality, the higher is the importance of nodes, which has a significant impact on the transmission of the information network. Details are shown in Table 3 and Table 4.

**TABLE 3:** List of national or regional collaborative network for anticancer natural products.

Rank	Frequency	Centrality	Country
1	13,323	0.16	People’s Republic of China
2	7,676	0.78	United States
3	2,727	0.02	India
4	2,686	0.02	South Korea
5	1,802	0.01	Taiwan, China
6	1,627	0.05	Japan
7	1,484	0.08	Germany
8	1,324	0.08	Italy
9	894	0.04	Brazil
10	872	0	Iran

**TABLE 4:** List of scientific research institutions in the cooperation network of anticancer natural products.

Rank	Frequency	Centrality	Institution
1	932	0.08	Chinese Academy of Sciences
2	732	0.03	China Medical University
3	494	0.02	Shanghai University of Traditional Chinese Medicine
4	417	0.02	Zhejiang University
5	405	0.01	China Pharmaceutical University
6	392	0.01	Shanghai Jiao Tong University
7	389	0.21	National Cancer Institute (NCI)
8	387	0.02	Kyung Hee University
9	362	0.01	Sun Yat Sen University
10	358	0.00	Nanjing University of Chinese Medicine

### Analysis of Subject Co-Occurrence

The co-occurrence analysis is mainly based on the “SC” field in the Web of Science text set. The “SC” field is the Web of Science designation for the subject area of each article. Using this analysis, we can understand the distribution of the subject areas involved in anticancer natural products.

**FIGURE 10:**
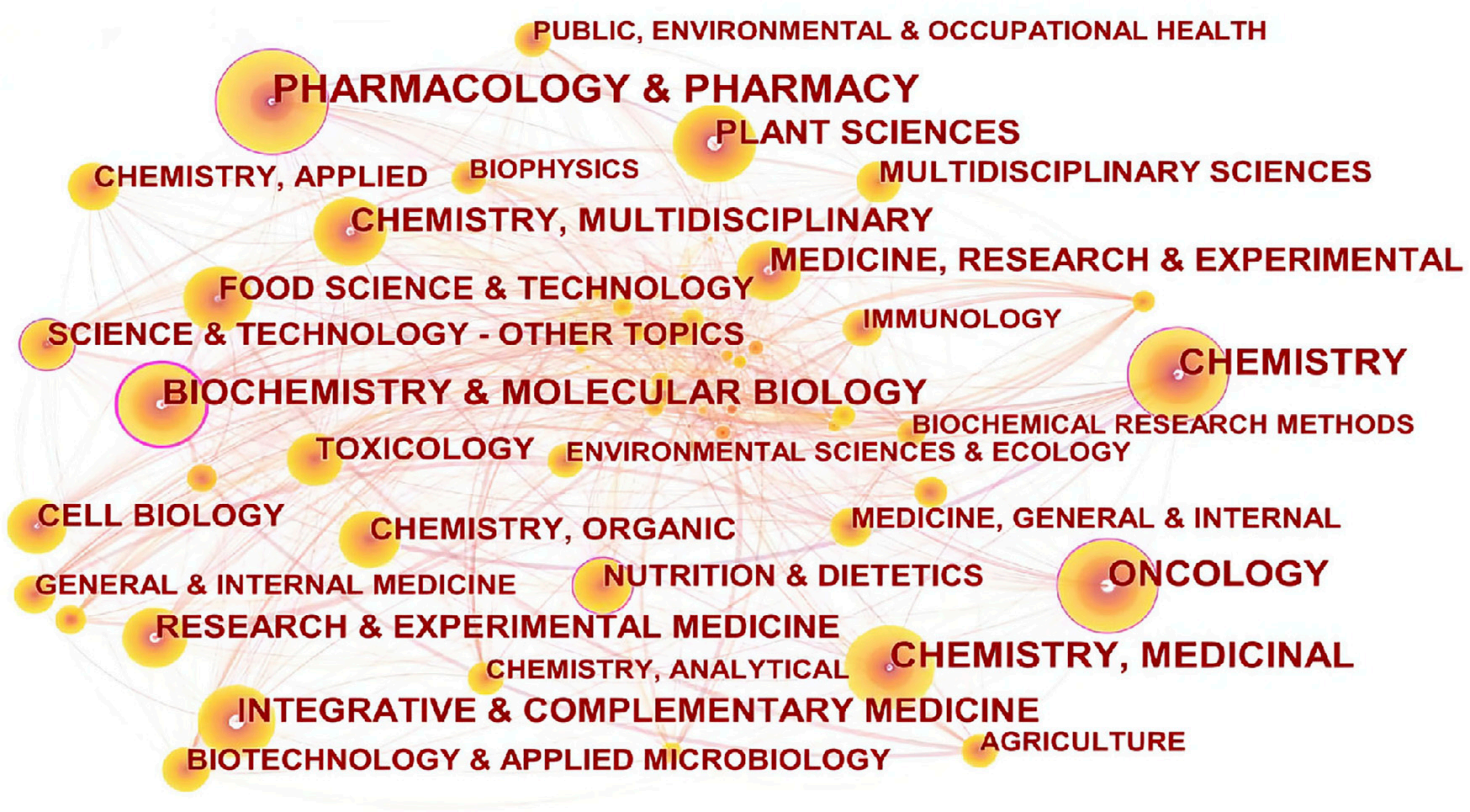
Subject co-occurrence network of scientific articles on anticancer natural products. Pharmacology and pharmacy has the highest co-occurrence frequency in the network; biochemistry and molecular biology has the highest centrality. The node size represents the number of articles in the subject area, the lines between the nodes represent the co-occurrence relationship, and the thickness of the lines represents the co-occurrence strength. The larger the node, the greater is the number of articles. Nodes with centrality greater than 0.1 are marked with a purple outer circle.

The running results showed that pharmacology and pharmacy, chemistry, and medicinal chemistry have high co-occurrence frequency in the network; based on the node centrality, it is shown that the research on anticancer natural products is mainly distributed in pharmacology and pharmacy, chemistry, oncology, biochemistry and molecular biology, and other subjects (Figure 10). Details are shown in Table 5.

**TABLE 5:** List of the subject co-occurrence network for anticancer natural products.

Rank	Frequency	Centrality	Category
1	11,490	0.18	Pharmacology and pharmacy
2	6,489	0.01	Chemistry and medicinal chemistry
3	6,369	0.13	Chemistry
4	5,459	0.16	Oncology
5	5,252	0.23	Biochemistry and molecular biology
6	4,022	0.01	Integrative and complementary medicine
7	3,332	0.02	Plant sciences
8	2,811	0.03	Chemistry and multidisciplinary
9	2,217	0.06	Research and experimental medicine
10	2,217	0.06	Medicine, research, and experimental

### Clustering Analysis of Keywords

This analysis can intuitively reveal the distance and similarity between keywords. CiteSpace divides each keyword into different clusters, according to their distances, and then, the distribution of research topics is observed in this field through the cluster labels of each cluster.

**FIGURE 11:**
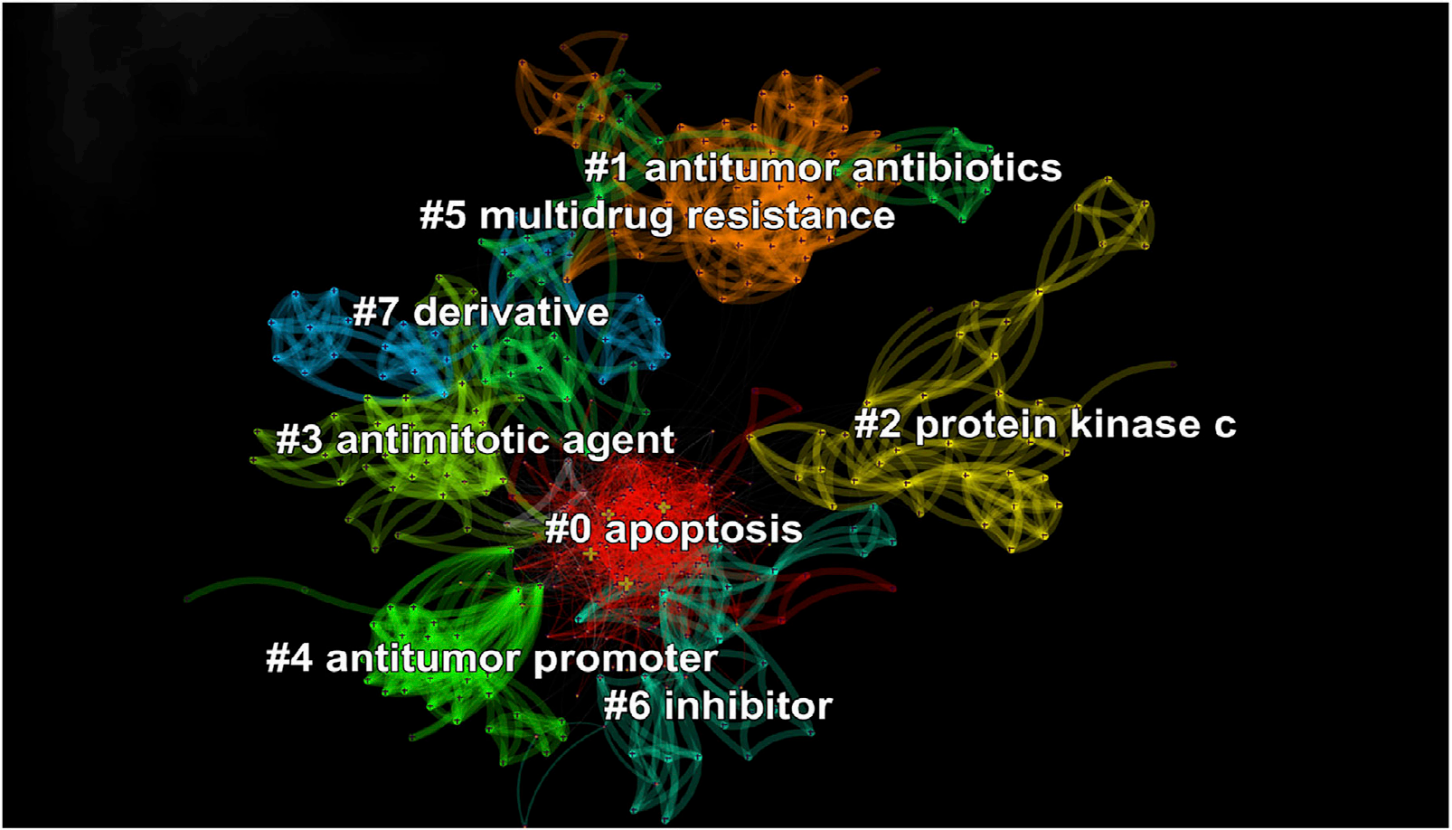
Keyword clustering network of scientific articles on anticancer natural products. Different colors mean different clusters. Anticancer natural products mainly focus on the themes “apoptosis,” “derivative,” “antitumor antibiotics,” “multidrug resistance,”, “antimitotic agent,” “antitumor promoter,” “inhibitor,” and “protein kinase C.”

According to the parameters of the running results, the S value (silhouette) is 0.9058, and the Q value (modularity) is 0.6446. The Q value greater than 0.3 means that the clustering structure is significant, and the S value greater than 0.7 indicates that the cluster members have high homogeneity; here, the clustering results are convincing. In this article, the first eight clusters were selected for display. The clustering results showed that the research on anticancer natural products mainly focuses on “apoptosis,” “derivative,” “antitumor antibiotics,” “multidrug resistance,” “antimitotic agent,” “antitumor promoter,” “inhibitor,” and “protein kinase C,” (Figure 11). The keyword information of each cluster is shown in Table 6.

**TABLE 6:** Detailed list of keyword clustering networks in scientific articles on anticancer natural products.

Cluster ID	Size	Silhouette	Keyword
#0	121	0.738	*Selaginella willdenowii*; nanoparticle; tumor necrosis factor; cell line; endothelial cell; green tea; Alzheimer’s disease; gene; metastasis; breast cancer; apoptosis; interferon gamma; cycle arrest; receptor; alternative medicine; drug discovery; migration; fruit; genistein; oxidase; NF-kappa B; antifeedant; target; risk; progression; therapy; drug resistance; mechanism; cancer cell line; factor alpha; p53; expression; downregulation; extract; enzyme; nitric oxide; women; antioxidant activity; tyrosine phosphorylation; NF-kappa B; messenger RNA; c-13 NMR; molecular docking; transcription; oxidative stress; prostate cancer; phytoestrogen; growth; carcinoma; survival; nitric oxide synthase; induction; *in vivo*; constituent; NF-kappa B; essential oil; gene expression; mulberry leaf extract; herbal medicine; cell; invasion; health; herbicide; establishment; carcinoma cell; breast cancer cell; cell cycle; *in vitro*; flavonoid; epithelial–mesenchymal transition; resistance; United States; polymorphism; proliferation; cell cycle arrest; medicinal plant; signaling pathway; chemoprevention; ovarian cancer; medicine; lipid peroxidation; antioxidant; anticancer activity; beta carotene; discovery; combination; cancer cell; cytochrome c; reductase; rat; colon cancer; natural product; inhibition; DNA; lung cancer; activation; hepatocellular carcinoma; inflammation; cancer prevention; tumor growth; traditional Chinese medicine; pathway; colorectal cancer; TNF alpha; cytotoxic biflavone; complementary; impact; prevention; induced apoptosis; network pharmacology; disease; prevalence; death; autophagy; phytochemical; kinase; estrogen; protein; phosphorylation; line; DNA damage
#1	59	0.986	directing group strategy; intramolecular cyclopropanation; antitumor ansamycin; d-glucose; Diels–Alder reaction; dimethyl sulfide complex; double asymmetric reaction; hydroxyl group; rearrangement; stereoselective synthesis; herbimycin A; chiral ligand; indole; conversion; coumarin; primary amine; enantioselective synthesis; sponge *Dysidea herbacea*; absolute stereochemistry; absolute configuration; stereoselective synthesis; macbecin I; ester; yuehchukene; hydroboration reaction; trisubstituted alkene; ansa compound; antitumor antibiotics; cyclohexene subunit; silyl ketene acetal; erythronolide A; *Lyngbya majuscula*; tetronolide; general approach; kijanolide; aldol condensation; N-methoxy-N-methylamide; antifertility agent; chlorothricolide; rainforest tree; tetrocarcin; diisobutylaluminum; tyrosine kinase activity; catalyzed asymmetric reaction; asymmetric synthesis; protecting group; antitumor antibiotics; microbial activity; ambruticin; anti-implantation agent; rifamycin biosynthesis; *Murraya*; Mosher method; benzostabase protecting group; decarboxylation; catalytic sodium tetrahydroborate; macbecins Ⅱ; stereoselectivity; stereoselective reducing agent
#2	39	0.956	scintillation proximity assay; jatropholone A; seedling; macrocyclic diterpene casbene; neoplasia; teleocidin; transgenic mice; aplysiatoxin; dioxirane; tumor promoter; stereochemistry; phorbol ester; biosynthesis; conformational states; growth factor alpha; hydrocarbon; pharmacophore; structural basis; calcium; ring system; chemistry; *Ricinus communis* L; cytospora; origin; protein kinase c; requirement; lyngbyatoxin A; model; precursor; debromoaplysiatoxin; stereoselective construction; highly inflammatory agent; reduction; alkoxy; diterpene; MPM (4-methoxybenzyl); cyclization; ingenane; ketone
#3	36	0.963	olefin metathesis approach; resistant; 2-methoxyestradiol; eleutherobin; curacin A; potent; podophyllotoxin; analog; binding; anticancer agent; antimitotic natural product; microtubule-stabilizing agent; *Combretum caffrum*; thiazoline; endogenous mammalian metabolite; curacin A; cell growth; combretastatin A4; colchicine; separation; cyanobacterium *Lyngbya majuscula*; cytotoxicity; taxol; antimitotic agent; prodrug; eleuthoside A; tubulin polymerization; antineoplastic agent; mulberry leaf extract; colchicine-binding site; 2-phenyl-4-quinolone; antitumor agent; tubulin; biological evaluation; epothilone A; colchicine site
#4	33	0.972	identification; *Salmonella typhimurium*; possible antitumor promoter; steroid; furocoumarin; *Lophira alata*; raji cell; grapefruit juice; 25-dihydrowithanolide D; quinone reductase induction; *Physalis philadelphica*; glycolipid; *Casimiroa edulis*; glyceroglycolipid; *Ruta graveolens* L.; *Citrus hystrix*; EB virus; withangulatin A; Dictamni radicis cortex; carcinogenesis; mutagenicity; cyanobacterium *Phormidium tenue*; oleanolic acid; root; resin glycoside; furoquinoline alkaloid; agent; ornithine decarboxylase; edible plant; antitumor promoter; cancer chemoprevention; Barr virus activation; *Withania somnifera*
#5	32	0.930	NMR spectroscopy; glycoside; glaucum; multidrug resistance; induced phospholipid metabolism; *giganteum*; *Didemnum* sp; soft coral; *Brodiaea californica*; epoxide; *schubertii*; phospholipid metabolism inhibition; cembranoid; camp phosphodiesterase; Na+/K + ATPase; alkaloid acronycine; natural product chemistry; inhibitory activity; tunicate; saponin; steroidal saponin; *Allium schubertii*; genus *Pseudopterogorgia*; dimethyl; whip *Pseudopterogorgia elisabethae*; metabolism; *Oncopeltus fasciatus*; scope; corpora allata; ultrastructure; *in vitro*; octocoral
#6	31	0.938	olefin; tumor; oxidation; alcohol; activated dimethyl sulfoxide; methionine aminopeptidase; immune checkpoint; palladium-catalyzed reaction; aldehyde; worker; mortality; trifluoroacetic anhydride; nitrone; skin tumor promotion; *Anabaena* flos-aquae; natural product; virus; Epstein–Barr virus; nucleophile; exposure; Epstein–Barr virus; risk factor; acylation rearrangement; folimycin (concanamycin A); angiogenesis; inhibitor; ATPase; phenoxy herbicide; Claisen rearrangement; curacin A; segment
#7	30	0.897	*vedeliana*; tumor virus; tumor cell line; tumor cell; toxicity; alveolar cancer; silver nanoparticle; shikalkin; reverse transcriptase; quinone; plant; *pilosa* Ledeb; phorbol ester bioactivity; NMR; New Caledonia; naphthoquinone derivative; metabolite; moricitrifone; *hanburyi*; green synthesis; Euphorbiaceae; drug; dimeric hydrolyzable tannin; derivative; delivery; cytotoxic activity; antitumor activity; anticancer; antitumor activity; acid

Detailed information on various clusters can be obtained using the “run batch mode” tab of the CiteSpace tool. The most relevant citer in the “apoptosis” cluster is an article by Abdolmohammadi et al. (2008). He first reported the anticancer activity of peachtree extract, persicus, and its possible mechanism of action. Persicus had a stronger inhibitory effect on T47D cells than RPMI control and doxorubicin. The most relevant citer in the “antitumor antibiotics” cluster is an article published by Martin et al. (1999) in the journal Tetrahedron, in which the asymmetric synthesis of herbimycin A was introduced. The most relevant study in the “multidrug resistance” cluster is an article by Limtrakul et al. (2007). In this study, the authors evaluated the ability of tetrahydrocurcumin (THC) to modulate MDR in tumor cells, and their experiments demonstrated that THC significantly inhibited the efflux functions of P-gp, MXR, and MRP1 and could prolong the MDR-reversal activity of curcumin *in vivo*. The most relevant citer in the “protein kinase C” cluster is an article by Kozikowski et al. (1997). Based on their modeling studies, they revealed how indolelactam V (ILV) and its analog benzalactam 15 bind to the CRD2 activator domain of protein kinase C (PKC).

The most relevant citer in the “antimitotic agent” cluster is an article published by Sanghai et al. (2014). He designed a novel antitubulin anticancer agent based on the pharmacodynamic characteristics of the natural product combretastatin A-4 (CA-4), and this synthesized analog could inhibit tubulin polymerization. Compared with CA-4, it showed stronger anticancer activity in breast and cervical cancer cell lines and had lower toxicity to normal cells. The most relevant citer in the “derivative” cluster is an article published by Patpi et al. (2012). The authors developed a lead drug candidate based on triazole-clubbed dibenzo [b, d] thiophene for the treatment of mycobacterial infections by molecular hybridization, highlighting the broad prospects of this novel structural modification tool for modifying pharmacodynamic properties. The most relevant citer in the “antitumor promoter” group is an article by Murakami et al. (1995), in which two glyceroglycolipids were isolated from *Citrus hystrix* and showed significant inhibitory effects on tumor promoter-induced Epstein–Barr virus (EBV) activation by carcinogenic experiments in ICR mice. In addition, the most relevant citer in the “inhibitor” cluster is an article by Goodwin et al. (1998). This article introduced a class of macrocyclic natural products, among which maytansine 1 is the most thoroughly studied. In terms of its activity, maytansine can inhibit the polymerization of tubulin, thus interfering with the function of microtubules. Therefore, its remarkable anticancer activity has attracted great attention of researchers.

### Burst Detection of Keywords

Keyword burst detection is mainly used to mine keywords with significant frequency fluctuations in a certain period of time. Through burst detection, the evolving trends in anticancer natural medicines from the macro to micro scale and from single to diversified sources can be found. This method is used to review or predict which branches of key technology will become hotspots or have continuous explosive trends in the future.

**FIGURE 12:**
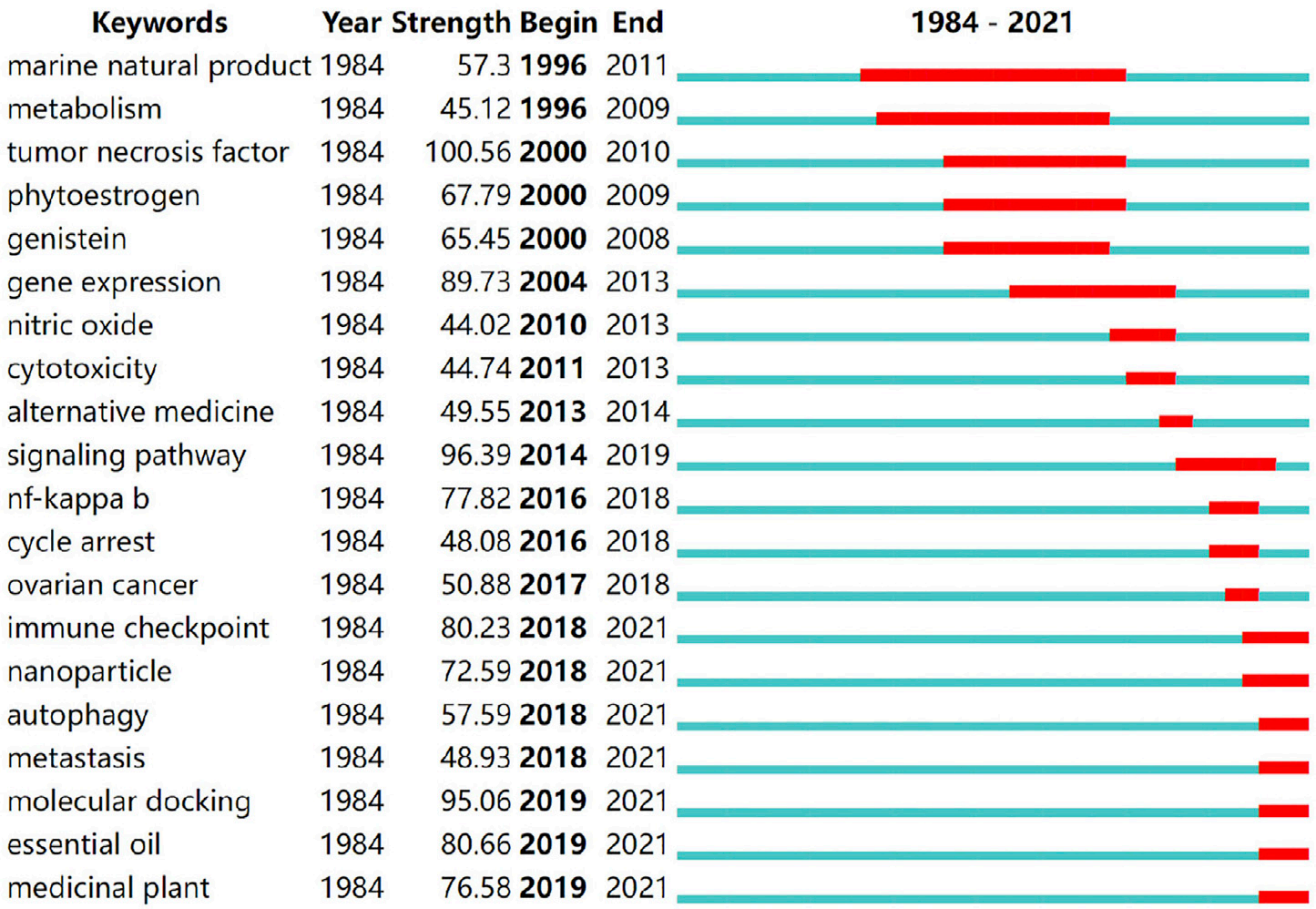
Details of keyword burst detection in scientific articles on anticancer natural products. The keywords “metastasis,” “immune checkpoint,” “medicinal plant,” “essential oil,” and “autophagy” maintain a large mutation intensity, and the mutation period continues to 2021. “Begin” represents the start time in keyword mutation, “End” represents the end year of the mutation, and “Strength” represents the keyword mutation strength. The larger the value, the greater is the frequency of keyword fluctuation during this time. The red area represents the time length in keyword mutation.

Based on the “burstness” function in CiteSpace, the bursty keywords were detected, and the default parameter settings were maintained. After detection, a total of 82 burst keywords were found in the network. To ensure the reliability of the detection results, this article uses the keyword mutation time as the basis for sorting, and the 20 nodes with the largest mutation intensity are selected (Figure 12). It can be seen from the figure that the keywords “metastasis,” “immune checkpoint,” “medicinal plant,” “essential oil,” and “autophagy” maintain a large mutation intensity, and the mutation period continues until this day.

Under the “immune checkpoint” node, Suzuki et al. (2021) developed the first double small-molecule immune checkpoint inhibitor CTLA-4 and PD-L1 using indole-like alkaloids. A study by Yuan et al. (2021) elucidated the mechanism by which licochalcone A (LCA) inhibits the IFN-gamma-induced immune checkpoint PD-L1 in lung cancer cells. Hwang et al. (2021) study found that as a local mucosal adjuvant, APS can enhance the antitumor effect of immune checkpoint inhibitors. The treatment of metastatic cancer is challenging due to complex histopathology and genetic variation. Under the “metastasis” node, Sp et al. (2018) and Yang et al. (2018) demonstrated the significant inhibitory effects of nobiletin and betulinic acid on the migration and invasion in breast cancer cells and human kidney cancer cells, respectively, showing the great potential of natural products to metastatic cancer.

Under “medicinal plant” node, Tripathi et al. (2019) *in vivo* experimental study demonstrated good antitumor effects and drug safety for plumbagin, and nanoembedded white flower dam formulation in particular overcame the obstacles of poor water solubility and bioavailability. Zehra et al. (2019) studied the anticancer activity of betulin 3 at different concentrations and introduced evidence of its great potential in the treatment of drug-resistant lung cancer. Under the “essential oil” node is an article by Abdalla et al. (2020) reporting a study on the anticancer activity of 1,8-cineole, the main component of EO. The cytotoxicity and proapoptotic properties of the compound were confirmed. Kubatka et al. (2020) evaluated the anticancer activity of cinnamon and found that its essential oil had a significant therapeutic effect in a breast cancer model. Maugeri et al. (2021) reported the anti-inflammatory and healing activities of bergamot essential oil, which was found to significantly reduce the level of tumor necrosis factor (TNF)-α and to have the potential for anti-edema and analgesia usages.

Under the “autophagy” node is an article by Teng et al. (2020), which reports a study of the underlying mechanisms of the anticancer properties of saponin polyphyllin VI, and the authors found that polyphyllin VI induced apoptosis and autophagy in non–small cell lung cancer, which makes it possible for polyphyllin VI to become a new drug for treatment of non–small cell lung cancer in the future. Wang L et al. (2020) conducted an in-depth study on the molecular mechanism and specific application of curcumin in inhibiting glioblastoma and found that curcumin inhibited proliferation by reducing the p-AKT/p-mTOR pathway and promoted apoptosis by increasing PTEN and p53 expressions.

## Discussion

From the trend of patent application every year, the overall number of patents in the field of anticancer natural products is on the rise. According to the application trend, the development of patent technology for natural anticancer drugs can be divided into five stages: 1967–1993 is the period of technology introduction, which represents the budding stage of technology; 1994–2005 is the first period of rapid growth; 2006–2012 is the period of technological adjustment, during which the number of patent applications was relatively stable, and technological innovation activities were relatively stable compared to the trend of the previous period. 2013–2015 is the second period of rapid growth, and patent applications increased significantly again until the technology saturation period when the number of patent applications and publications declined to a certain extent. In terms of the annual publication statistics, the number of scientific articles published in this field is increasing year by year, indicating that scientific research and innovation activities are increasingly active.

In terms of the distribution of patented technologies, those of anticancer natural medicines are mainly concentrated in the field of medicine and health and also distributed in other fields such as biochemistry, microbiology, mutation or genetic engineering, organic chemistry, and food. The key areas of the distribution of scientific articles are in the subject areas of pharmacology and pharmacy, chemistry, oncology, biochemistry, and molecular biology.

Based on the results of cluster analysis, we sorted out the natural products according to the different anticancer mechanisms (Table 7). Some natural products, such as curcumin, resveratrol, artesunate, and gallic acid, exert anticancer effects by inducing cell apoptosis. Theranekron D6 (Tosun et al., 2021) obtained from spider venom and gaillardin (Roozbehani et al., 2021) isolated from *Inula oculus-christi* have attracted great attention due to their significant proapoptotic properties, and therefore, they may be powerful candidates for inducing apoptosis in the future. Camptothecin, curcumin (Rowaiye et al., 2021), isoquinoline alkaloids of *Chelidonium majus* (Noureini et al., 2017), amentoflavone (Revikumar, 2021), and emodin (Liu et al., 2019), are effective telomerase inhibitors. Some natural compounds, such as fucoidan (Yang et al., 2021), myricetin (Ghassemi-Rad et al., 2018), bergapten (Xie et al., 2018), and atractylenolide I (Xu et al., 2021), have good immunomodulatory effects so that they have great potential as candidates for immunotherapy in the future. In addition, tanshinone IIA analog (Huang C et al., 2021), kolaflavanone (KLF) (Alrazi et al., 2021), eugenol (Abdalla et al., 2020), and millepachine (Yan F et al., 2021), considered as potentially potent microtubule inhibitors, inhibit cell growth by interfering with the formation of microtubules. Epigallocatechin-3-gallate (EGCG) (Chen et al., 2019), lupeol (LUP) (Bociort et al., 2021), saikosaponin A (Cheng and Ying, 2021), and ononin (Gong et al., 2021) can significantly supress tumor angiogenesis.

**TABLE 7:** List of anticancer natural product classifications based on pathways of action.

Mode of action	Natural product	Compound activity	Author
Apoptosis inducer	Ursolic acid	Degrades ferritin by activating autophagy and induces intracellular overload of ferrous ions, leading to ferroptosis	Tang et al. (2021)
	Propolis	Induction of apoptosis through bax, bcl-2, p53, and caspase-3 pathways; angiogenesis targets are the vascular endothelial growth factor receptor and protein kinase A.	Mehany et al. (2022)
		Stage 1: activates Nrf2 and reduces ROS; stage 2: inhibits the constitutive expression of Nrf2 in AsT and promotes ROS and apoptosis	Dang et al. (2021)
	Curcumin	Curcumin analogs (CA-5f) decreases uPA protein levels by upregulating PAI-1.	Kim and Moon, (2022)
		PLGA-Cur-NP treatment synergistic with I kappa B alpha overexpression enhances the apoptosis of PC cells by suppressing NF-kappa B pathway activation	Guo et al. (2021)
	Resveratrol	Combination of resveratrol and curcumin showed stronger cytotoxicity, especially related to the induction of stronger endoplasmic reticulum stress and the upregulation of the pro-death UPR molecule CHOP.	Arena et al. (2021)
		Promotes apoptosis-related protein caspase-3 to activate poly-ADP-ribose polymerase and cleavage of caspase-3 and reduces survivin protein levels in a dose-dependent manner	Ma et al. (2021)
	Gaillardin	Inhibits NF-kappa B activation and subsequently downregulates genes regulated by NF-kappa B	Roozbehani et al. (2021)
	Theranekron D6	Increases the expression of Cas-9 and the mRNA and protein expression ratio of bax/bcl-2	Tosun et al. (2021)
Antiangiogenic and antimetastatic agents	Epigallocatechin-3-gallate (EGCG)	Significantly reduces the level of HUVECs endothelin/pSmad1 and inhibits angiogenesis by downregulating VEGF.	Chen et al. (2019)
	Lupeol (LUP)	Interferes with the angiogenesis process by reducing the formation of new blood vessels	Bociort et al. (2021)
	Saikosaponin A	Regulation of the angiogenesis-related VEGFR2/Src/Akt pathway and expression of epithelial–mesenchymal transformation (EMT)-related proteins inhibit SK-N-AS invasion and migration	Cheng and Ying, (2021)
	Ononin	Inhibits HUVEC migration and invasion induced by vascular endothelial growth factor (VEGF)	Gong et al. (2021)
	Berberine	Mitochondrial function is impaired by impairing mitochondrial membrane potential and mitochondrial complex I, resulting in the selective elimination of Mdr1p overexpressed *C. albicans* cells	Tong et al. (2021)
	Quercetin	Downregulates the expression of the glutamine transporter solute carrier family 1, member 5 (SLC1A5) in SW620/Ad300 cells	Zhou et al. (2020)
Multidrug resistance reversal agents	Babaodan (BBD)	Reverses the MDR and induces apoptosis and autophagy of SGC7901/DDP cells and inhibits the PI3K/AKT/mTOR pathway activity	Zhao et al. (2021)
	Dihydromyricetin (DMY)	Restores chemosensitivity (OXA and VCR) by inhibiting both the MRP2 expression and its promoter activity in HCT116/OXA and HCT8/VCR cell lines, inhibits the expression of NF-Kappa B/P65, and reduces the translocation of NF-Kappa B/P65 to the nuclear silencing Nrf2 signal	Wang et al. (2021)
	Fucoidan	(TCR)/CD3 complex, enhances the TCR-mediated signaling pathway, and cooperates with the JAK-STAT pathway to stimulate T-cell activation	Yang et al. (2021)
Immunomodulatorsr	Myricetin	Inhibits cell proliferation and reduces the synthesis of interferon, interleukin (IL)-2, IL-4, and IL-17 associated with different T-helper cell subpopulations	Ghassemi-Rad et al. (2018)
	Bergapten	Promotes T-cell proliferation and upregulates IFN-γ and IL-4 cytokines in aging mice	Xie et al. (2018)
	A. membranaceus polysaccharides (APS)	Intranasal treatment of APS activated DCs, which further stimulated natural killer (NK) and T cells in the mLN.	Hwang et al. (2021)
	Atractylenolide I (ATT-I)	Binding of ATT-I to PSMD4 enhances the antigen processing activity of the immune proteasome, leading to the enhancement of MHC-I-mediated antigen presentation on cancer cells.	Xu et al. (2021)
	Kolaflavanone (KLF)	Inhibits both the basal and microtubule-activated ATPase activities of Eg5	Alrazi et al. (2021)
	Tanshinone IIA analog	The structurally modified compound 2F binds to the tubulin colchicine site, inhibits tubulin assembly, and destroys the normal formation of the microtubule network.	Huang H et al. (2021)
Microtubule inhibitor	Eugenol	Autophagy was induced by the upregulation of microtubule-associated protein 1 light chain 3 (LC3) and the downregulation of nuclear pore protein 62 (NUp62)	Abdalla et al. (2020)
	Millepachine (MIL-1)	Interferes with the balance of tubulin-microtubule dynamics, irreversibly binding to tubulin; cell cycle arrest occurred in the G2/M phase, and apoptosis was induced by activation of the caspase-3 activity and accumulation of reactive oxygen species (ROS)	Yan J et al. (2021)
	Camptothecin	Selective inhibition of topoisomerase ⅰ, by binding topoisomerase Ⅰ to the complex formed by DNA to prevent tumor cell DNA replication and RNA synthesis	Pourquier and Lansiaux, (2011)
Topoisomerase inhibitor	Scaffold-hopped flavones	Synthesized based on the strategy of scaffold-hopping, and it has more significant inhibitory activity against human TopoⅡα	Priyadarshani et al. (2016)
	Oxocrebanine	Regulation of TopoⅠ, Ⅱα, and DNA damage-related proteins	Yu et al. (2021)
	Evodiamine derivatives	Anticancer and antihepatic fibrosis *via* blocking topoisomerase, NF-κB, TGF-β/HGF, and Smad2/3	Fan et al. (2021)
Telomerase inhibitor	Isoquinoline alkaloids (*Chelidonium majus*)	Strongly interacts with telomere sequence G-quadruplex; telomerase activity was inhibited by substrate isolation	Noureini et al. (2017)
	Camptothecin	Camptothecin, like curcumin, binds to 10 functional domains of hTERT. Camptothecin has stronger molecular stability and telomerase inhibitor activity	Rowaiye et al. (2021)
	Curcumin		
	Amentoflavone	Telomerase activity is blocked by the formation of Quadruplex DNA at the ends of telomeres.	Revikumar, (2021)
	Emodin	G4 structure stabilizes *in vitro* and induces telomere dysfunction	Liu et al. (2019)
	β-escin	β-escin and the cardiac glycosides inhibit ECM production in mesothelial cells and fibroblasts	Lengyel et al. (2021)
Tumor microenvironment regulator	Icaritin	Increased infiltration of CD8^+^ T cells in the TME; CD8^+^ T-cell infiltration in the TME was promoted by the downregulation of immunosuppressive cytokines (TNF-α, IL10, and IL6) and the upregulation of chemotaxis (CXCL9 and CXCL10)	Huang C et al. (2021)

Natural products such as berberine (Tong et al., 2021), quercetin (Zhou et al., 2020), and dihydromyricetin (DMY) (Wang Z et al., 2020) can reverse the multidrug resistance of tumor cells. Beta-escin (Lengyel et al., 2021) and icaritin (Huang H et al., 2021) exert anticancer effects by regulating and improving the tumor microenvironment. A novel strategy in systematic pharmacology integrates active compound screening, target prediction, network pharmacology analysis, and oncoimmune phylogeny to identify the potential active components of natural products. This approach provides an important method for enabling us to further explore the multiple pharmacological mechanisms of natural products targeting the tumor microenvironment (Huang C et al., 2021). The main sources of topoisomerase inhibitors in the past were compounds such as camptothecin (Pourquier and Lansiaux, 2011) and podophyllotoxin (Hong et al., 2016). However, previous topologically oriented drugs, such as camptothecin, had great limitations due to their clinical toxicity and side effects. In recent years, the significant inhibition of topoisomerase isolated from natural products such as evodiamine derivatives (Fan et al., 2021) and oxocrebanine (Yu et al., 2021) has raised great interest. The low cytotoxic natural products found in coral-derived fungi (Xin et al., 2017) have great potential in inhibiting Topo I, which opens new possibilities for the development of novel nontoxic topoisomerase inhibitors.

In addition, the unexpected activity of new scaffolds for natural products compared to older molecules is also of great interest. Priyadarshani et al. (2016) first reported the scaffold-hopping strategy of flavones and isoflavones and showed that those scaffold-hopping analogs had significant/enhanced Topo II *α* inhibition and cytotoxic properties except for the similar functions to parent flavones/isoflavones, providing a potential strategy for drug discovery. Wang L et al. (2020) designed a novel antitumor indolopyrazinoquinazolinone scaffold based on scaffold skipping and verified its superior tumor-suppressive activity compared to the original compounds, opening up a broader road for development of new high-efficiency and low-toxic anticancer molecules in the future.

Currently, the development of medicinal plants and the research on the anticancer mechanism of natural active products are still hotspots; in particular, immune checkpoint, essential oil, metastatic cancer, and other related topics deserve attention. It is worth noting that for a long time, we have carried out a large number of preclinical studies on the anticancer mechanisms of natural drugs or traditional Chinese medicine, but there is still a great lack of in-depth integration of these research results with clinical theories of traditional Chinese medicine. Theories in traditional Chinese medicine, such as “holistic concept,” “same treatment for different diseases,” and “survival with cancer,” have gradually been accepted by the Western concept of cancer treatment.

“Restraining excessiveness to acquire harmony,” one of the basic theories of traditional Chinese medicine, is a high-level summary of the homeostasis balance under physiological conditions, the compensatory self-healing mechanism under pathological conditions, and the rule of treatment effect under treatment purposes. Based on the complex physiological and pathological conditions in immune T cells and the theory of “restraining excessiveness to acquire harmony,” Cai et al. (2022) proposed that long-term survival with tumors might be achieved by suppressing cancer cells to replenish vitality, correcting bodily deficiencies, and promoting the homeostatic balance by restraining excessiveness to acquire harmony. Li (2018) demonstrated that Guizhi decoction had good efficacy on cardiac autonomic nerve remodeling and coronary microvascular disease in clinical and animal experiments, enhancing the inheritance and innovation in the TCM theory.

In the TCM theory, the tumor pathogenesis is actually the imbalance between “Zhengxu” and “Duxie”. Li Q. W et al. (2021) explored the relationship between “Fuzheng” and “Quxie” in the treatment of tumor in TCM and confirmed the superiority of “Fuzheng” and “Quxie”. Yan F et al., 2021 have evaluated the therapeutic effect of Yangyin Fuzheng Jiedu Prescription (YFJP) on H22 tumor-bearing mice and found that its antitumor mechanism is related to alleviating T-cell exhaustion and immunosuppression, enhancing our understanding for “Fuzheng” and “Quxie” treatment. Based on YIQIHUOXUEJIEDU theory, Li J. X et al. (2021) explored the antitumor potential of SANT (astragaloside IV, alpha-solanine, neferine, and 2,3,5,6-tetramethylpyrazine derived from *Astragalus mongholicus*, *Solanum nigrum* Linn, Lotus plumule, and *Ligusticum*, respectively) in heparanase-related triple-negative breast cancer (TNBC) *in vitro* and *in vivo*, showed a promising candidate of herbal compounds, and provided novel strategies for using natural compounds to achieve an optimized effect. In addition, the unique TCM treatment features of “same treatment for different diseases” (Hu et al., 2022) and “Meridian induction theory” (Li et al., 2022) have great guiding significance for us to develop new anticancer drugs.

### Limitations

Due to the heavy workload of retrieving the patent literature and scientific articles in this study, other databases beyond this study are not considered, so there may be omissions in data. In addition, patent analysis is limited by research tools and a lack of in-depth mining of the full text of patents. These problems may be further improved by constantly updating research methods in the future.

## Conclusion

In summary, based on the patent literature and scientific articles, this article described the research status and hotspots of anticancer natural products for the first time and drew the following conclusions: 1) the technology of anticancer natural products was introduced earlier, but the later development momentum was insufficient; 2) scientific research activities are relatively closed, and technical exchanges need to be strengthened; 3) the development of medicinal plants, marine natural products, and the related anticancer mechanism are the current research hotspots. In particular, essential oils and immune checkpoint inhibitors have attracted great attention; and 4) TCM theories such as “restraining excessiveness to acquire harmony,” “same treatment for different diseases,” “Meridian induction theory,” and “Fuzheng Quxie” have important guiding significance for the study of anticancer mechanisms and discovery of new anticancer drugs.

## Data Availability

The raw data supporting the conclusions of this paper are provided by the authors.
